# Experience With Transcanal Endoscopic Ear Surgery and Preoperative Imaging in the Case of Persistent Stapedial Artery

**DOI:** 10.7759/cureus.73337

**Published:** 2024-11-09

**Authors:** Yoshiyuki Sasano, Yuichiro Yaguchi, Izumi Koizuka, Manabu Komori

**Affiliations:** 1 Department of Otolaryngology, St. Marianna University School of Medicine, Kawasaki, JPN

**Keywords:** cta – computed tomographic angiography, persistent stapedial artery, stapes surgery, tees, transcanal endoscopic ear surgery

## Abstract

The persistent stapedial artery (PSA) is an exceedingly vascular anomaly that can lead to hearing loss or pulsatile tinnitus, yet its preoperative diagnosis is often challenging. We report the case of a 24-year-old woman with bilateral PSA and stapes ankylosis who presented with progressive bilateral mixed hearing loss. The patient was initially diagnosed with bilateral congenital stapes ankylosis and stapes surgery was performed on the left side using transcanal endoscopic ear surgery (TEES). Intraoperative observation of the tympanic cavity revealed the presence of a persistent stapedial artery. The stapes surgery was completed successfully while preserving the stapedial artery, resulting in postoperative hearing improvement. Subsequently, surgery was planned for the right ear. Suspecting the possibility of PSA, we performed a contrast CT scan; additionally, we prepared subtraction images and CT angiography. These images strongly suggested the presence of the PSA on the right side as well. This case highlights the superior surgical visualization provided by TEES and underscores the effectiveness of advanced imaging techniques in diagnosing PSA.

## Introduction

The stapedial artery develops from the internal carotid artery during the fetal period and involutes around the 10th week of fetal age [1]. However, in rare cases, the artery persists, resulting in a condition known as persistent stapedial artery (PSA). It is often found incidentally when exploratory tympanotomy is performed for idiopathic conductive hearing loss or when a stapes surgery is performed after the diagnosis of otosclerosis. PSA frequently accompanies stapes ankylosis, necessitating surgical intervention [2]. The condition presents distinct challenges in the presence of PSA compared to typical otosclerosis. First, preoperative diagnosis is often difficult [3]. Second, manipulation of the PSA could cause profuse bleeding [4]. In this report, we discuss imaging modalities that may be instrumental in the preoperative diagnosis of this rare anomaly and present a case of PSA successfully managed with transcanal endoscopic ear surgery (TEES).

## Case presentation

A 24-year-old woman presented with a history of bilateral hearing loss since childhood, for which she had been using bilateral hearing aids. Her father also had a family history of hearing loss. The patient had noticed a progression of the hearing loss but reported no tinnitus. Suspecting an ossicular malformation, she was referred to our hospital.

**Figure 1:**
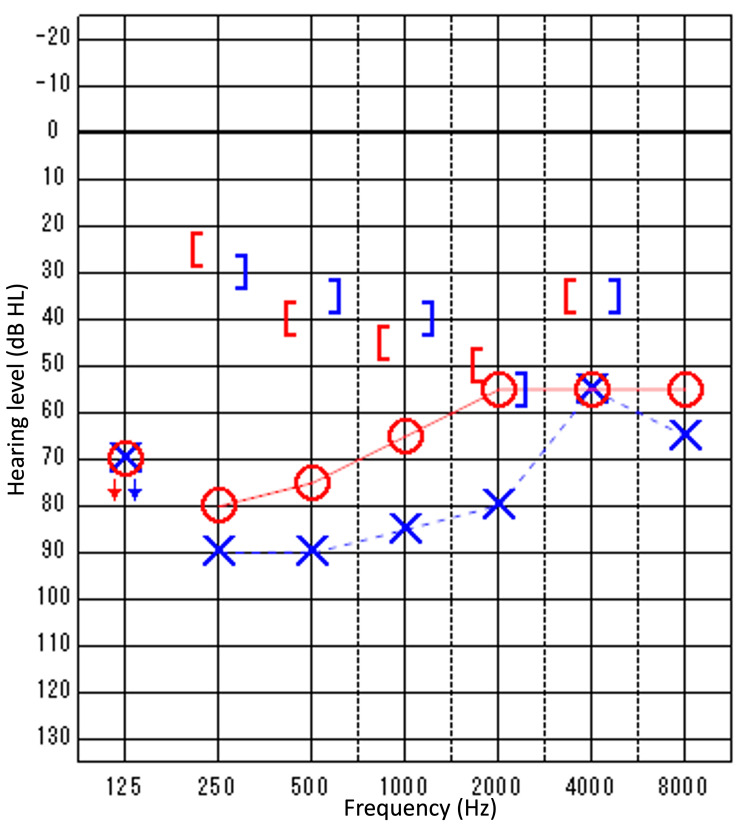
Preoperative audiograms. PTA indicated mixed hearing loss on the right side at 65 dB and on the left side at 85 dB using the four-frequency average method. Circle and Red Solid line: Right Air Conduction; Cross and Blue Dotted line: Left Air Conduction; Down Arrow: Scale Out; Left Square Bracket: Right Masked Bone Conduction; Right Square Bracket: Left Masked Bone Conduction PTA: Pure Tone Audiometry

**Figure 2:**
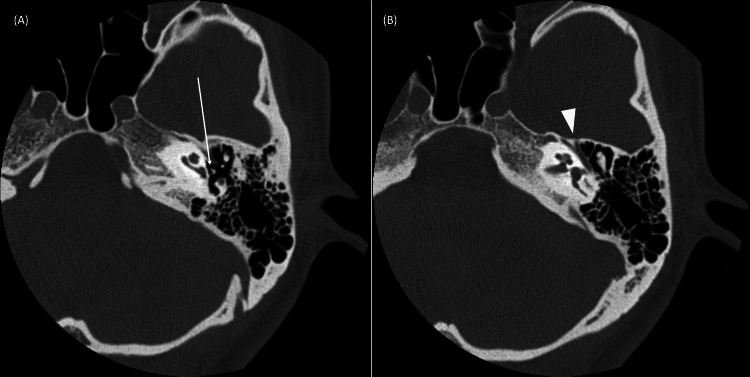
Temporal horizontal bone CT scan of the left temporal bone. (A) The white arrow indicates a thicker anterior limb of the stapes. (B) The arrowhead indicates an abnormal facial nerve pathway.

Pure tone audiometry (PTA) indicated bilateral mixed hearing loss on the right side at 65 dB and on the left side at 85 dB using the four-frequency average method (Figure 1). The acoustic reflex was unresponsive bilaterally. Temporal bone CT showed a thicker anterior limb of the stapes and an abnormal facial nerve pathway (Figure 2). 

**Figure 3:**
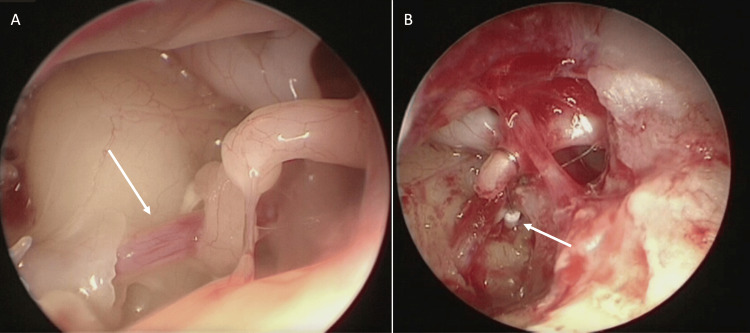
Intraoperative findings. (A) The white arrow indicates the stapedial artery running between the anterior and posterior limbs of the stapes. (B) A wire piston (white arrow) was inserted into the footplate and connected with the long limb of the incus.

Based on the above results, we diagnosed her with congenital stapes ankylosis and decided to perform stapes surgery using TEES. After elevating the tympanomeatal flap and observing the tympanic cavity, we identified the stapedial artery running between the anterior and posterior limbs of the stapes, in addition to stapes ankylosis and limb malformation (Figure 3A). The incus-stapes joint was then dissected, and the posterior limb of the stapes was cut with a nipper. Hemorrhage from the stapedial artery was noted during the procedure, but hemostasis was successfully achieved by applying compression with a cotton ball soaked in adrenaline (5000×). The oval window was subsequently opened, and a wire piston was inserted while preserving the stapedial artery and connecting it to the long process of the incus (Figure 3B). No facial palsy was observed postoperatively.

**Figure 4:**
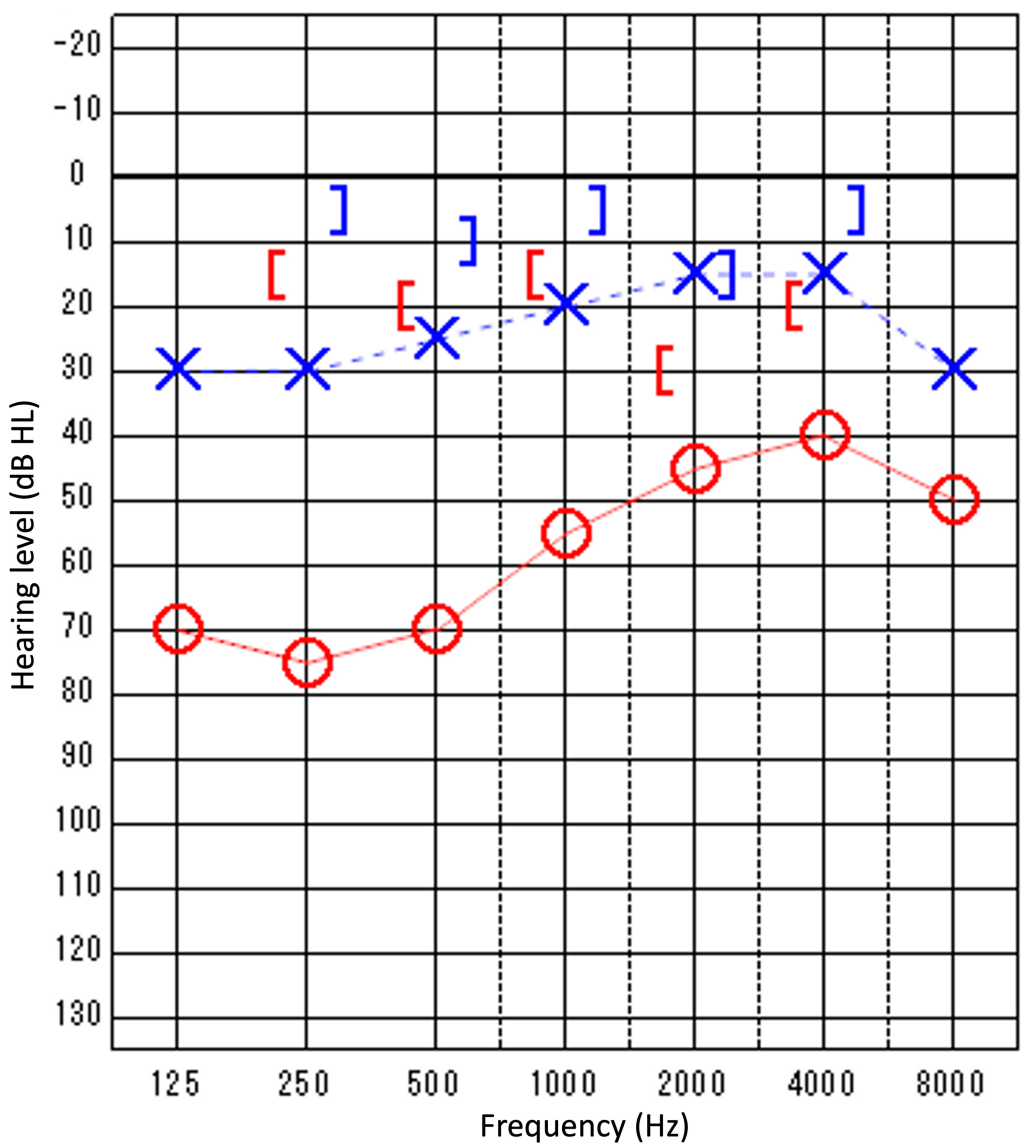
Postoperative audiograms. Postoperative hearing indicated a hearing level of 20 dB and decreased A-B Gap in 6 months after surgery than preoperative. Circle and Red Solid line: Right Air Conduction; Cross and Blue Dotted line: Left Air Conduction; Left Square Bracket: Right Masked Bone Conduction; Right Square Bracket: Left Masked Bone Conduction

On the first postoperative day, the patient reported dizziness and was found to have direction-fixed, right-beating nystagmus by positional nystagmus. She was treated with 300mg of hydrocortisone for three days. The dizziness started to improve by the second postoperative day, and the nystagmus resolved by the third postoperative day. The patient was discharged on the sixth postoperative day. Six months after surgery, postoperative audiometry indicated a hearing level of 20 dB in the left ear (Figure 4).

**Figure 5:**
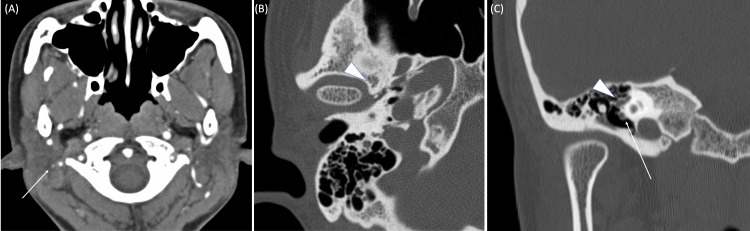
Axial and coronal contrast-enhanced CT scan of the right temporal bone. (A) The white arrow indicates a blood vessel from the branch of the right external carotid artery. (B) The white arrowhead indicates a defecting of the spinous foramen. (C) The white arrowhead indicates a vascular structure of the promontory. The white arrow indicates an enlargement from the facial nerve tympanic segment to the labyrinthine segment.

At the patient's request, we planned surgery for the right ear and performed temporal bone contrast-enhanced CT. It showed that a blood vessel coming from the branch of the right external carotid artery continued from the jugular foramen to the inside of the temporal bone (Figure 5A). It also showed a defect of the spinous foramen (Figure 5B), vascular structure of the promontory and enlargement from the facial nerve tympanic segment to the labyrinthine segment (Figure 5C). 

**Figure 6:**
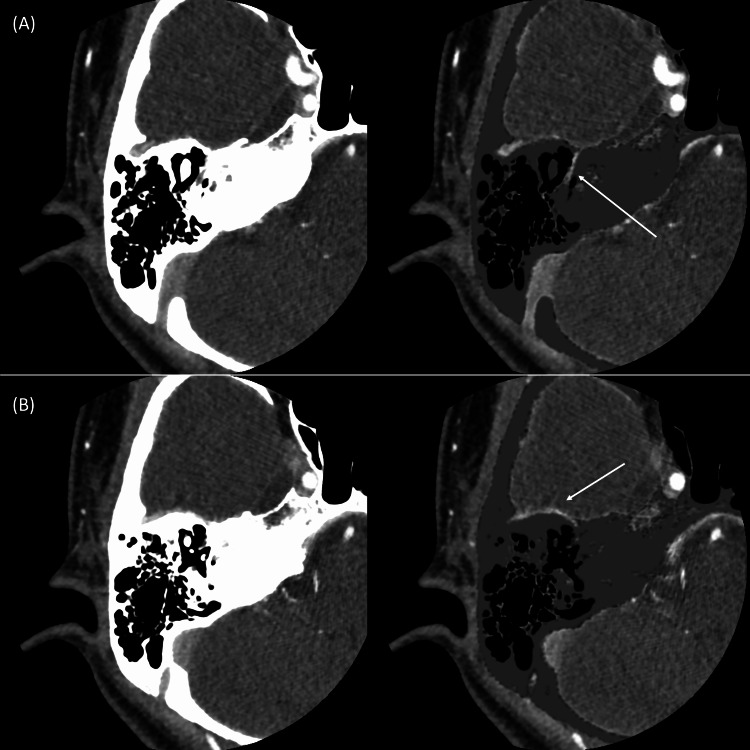
Subtraction images of right temporal bone CT. The right side is a subtraction image and the left side is an image of the soft tissue window. The slices are the same on both sides. A vascular structure runs around the stapes (A: white arrow) and is connected to the middle meningeal artery (B: white arrow).

**Figure 7:**
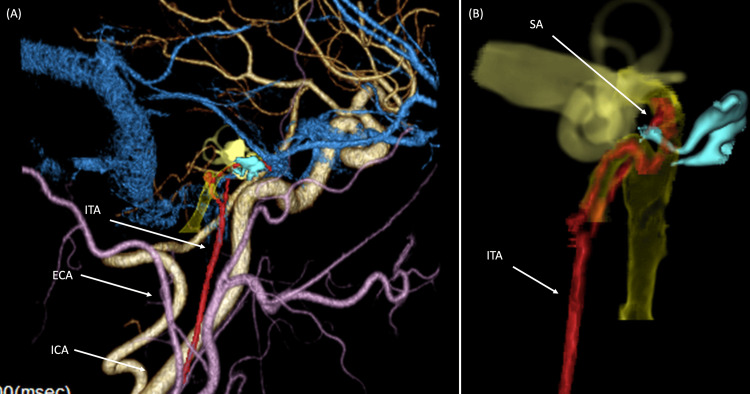
CTA images. (A) This image shows the location of the ear ossicles and surrounding vascular structures. The inferior tympanic artery doesn't divide from the internal carotid artery but the external carotid artery. (B) The stapedial artery running between the anterior and posterior limbs of the stapes. ITA: Inferior Tympanic Artery; ICA: Internal Carotid Artery; ECA: External Carotid Artery; SA: Stapedial Artery Image credits: Yoshiyuki Sasano

We prepared subtraction images and CT angiography (CTA) scans based on contrast-enhanced CT scans. The subtraction images showed the vascular structure running around the stapes and connected to the middle meningeal artery can be more clearly identified through the comparison of the soft conditions of contrast-enhanced CT (Figure 6). CTA showed that the stapedial artery was found running between the anterior and posterior limbs of the stapes (Figure 7). These findings strongly suggest the presence of the PSA on the right side as well. Surgery for the right ear is scheduled for the near future.

## Discussion

The PSA is very rare, accounting for 0.02-0.48% of cases [5]. The stapedial artery, a feeding vessel for the development of stapes, is generated as a branch of the internal carotid artery and connected to the middle meningeal artery. When the maxillary artery, or a branch of the carotid artery, starts to supply blood to the middle meningeal artery, the stapedial artery becomes atrophic and disappears [6]. Since PSA often accompanies stapes ankylosis and presents mixed hearing loss, stapes surgery is often required. However, bleeding from the stapedial artery due to intraoperative manipulation may make the surgery difficult [7]. In the present case, we performed stapes surgery utilizing TEES while successfully preserving the stapedial artery. The use of TEES provided an optimal surgical field, allowing precise visualization of the stapedial artery during wire insertion. Although there are no prior reports in the literature of stapes surgery being performed with TEES in the presence of a stapedial artery, TEES has potential efficacy in such cases. In a previous report, ligation of the stapedial artery for the prevention of bleeding may cause facial palsy [8]. It is probably because the upper petrosal branch of the middle meningeal artery, which feeds the facial nerve, becomes ischemic due to the ligation of the stapedial artery connected to the middle meningeal artery [8]. When the posterior limb of the stapes was cut, bleeding from the stapedial artery was observed, but hemostasis was achieved by appropriate compression. Despite of one-handed operation inherent in TEES, the procedure was completed successfully with preservation of the stapedial artery, and no postoperative facial palsy was observed.

Preoperative diagnosis of PSA is challenging. Consistent with previous observations, we initially suspected congenital stapes ankylosis and only identified the PSA intraoperatively. Contrast-enhanced CT is useful for preoperative diagnosis. Typical findings include (1) a small duct structure branching from the internal carotid artery, (2) a defect of the spinous foramen, (3) enlargement of the horizontal part of the facial nerve, and (4) vascular structure of the tympanic promontory [9]. Our patient showed the typical findings of (2)-(4). Regarding finding (1), continuity was observed not from the internal carotid artery but from the external carotid artery, which was considered to be the pharyngo-stapedial artery pattern among the types of PSA proposed by Hitier [8]. Angiography is useful as a diagnostic procedure [10], although it may be limited in evaluating the spatial relationship between the stapedial artery and surrounding structures such as the ossicles. In this case, we prepared subtraction images and CTA scans based on contrast-enhanced CT scans to better visualize the stapedial artery. Subtraction imaging enhances lesion visibility by eliminating hyperintense regions, such as bone or stents, that may obscure vascular structures on CT scans. This technique is used for the evaluation of coronary arteries in patients with stent-graft insertion [11] and for the evaluation of skull base invasion by nasopharyngeal carcinoma [12]. CTA is useful in revealing the pathway of each blood vessel and the positional relationship of the surrounding structures in 3D imaging. These imaging modalities may hold significant value in the preoperative diagnosis of PSA in the future.

This is a case report of a single case, and further studies with multiple cases and long-term outcomes are needed for the generalization of the utility of TEES and advanced imaging techniques in managing PSA.

## Conclusions

This case report provides valuable insights into the management of PSA, particularly regarding stapes surgery and advanced imaging techniques. We emphasize the utility of the TEES technique and the importance of imaging studies in diagnosing PSA. Given the difficulty in preoperatively diagnosing PSA, we recommend considering contrast-enhanced CT, as well as the preparation of subtraction or CTA images, when PSA is suspected. Although this case contributes significantly to the understanding of PSA, further research involving multiple cases and long-term outcomes is necessary to validate and generalize these findings.
